# Heterozygous variant in *WNT1* gene in two brothers with early onset osteoporosis

**DOI:** 10.1016/j.bonr.2021.101118

**Published:** 2021-08-18

**Authors:** Christie G. Turin, Kyu Sang Joeng, Staci Kallish, Anna Raper, Stephanie Asher, Philippe M. Campeau, Amna N. Khan, Mona Al Mukaddam

**Affiliations:** a Department of Medicine, Division of Endocrinology, Diabetes and Metabolism, Perelman School of Medicine, University of Pennsylvania, 3400 Civic Center Blvd., 4th floor, Philadelphia, PA 19104, USA; b Mckay Orthopaedic Research Laboratory and Department of Orthopaedic Surgery, Perelman School of Medicine, University of Pennsylvania, 3450 Hamilton Walk, Philadelphia, PA 19104, USA; c Department of Medicine, Division of Translational Medicine and Human Genetics, Perelman School of Medicine, University of Pennsylvania, 3400 Spruce Street, 5100 Silverstein, Philadelphia, PA 19104, USA; d Department of Pediatrics, University of Montreal, Montreal, QC, Canada; e Section of Endocrinology, The Corporal Michael J. Crescenz VA Medical Center, 3900 Woodland Ave, Philadelphia, PA 19104, USA; f Departments of Medicine and Orthopaedic Surgery, The Center for Research in FOP and Related Disorders, Perelman School of Medicine, University of Pennsylvania. 3737 Market St., 3rd floor, Philadelphia, PA 19104, USA

**Keywords:** ACMG, American College of Medical Genetics, BMD, Bone mineral density, DXA, dual-energy X-ray absorptiometry, VUS, variant of unknown significance, WNT1 pathway, Genetic variant, Early osteoporosis, Fragility fractures

## Abstract

Osteoporosis is a multifactorial disorder characterized by low bone mass and strength, leading to increased risk of fracture. The WNT pathway plays a critical role in bone remodeling by enhancing osteoblastic differentiation, which promotes bone formation, and inhibiting osteoclastic differentiation, decreasing bone resorption. Therefore, genetic alterations of this pathway will lead to impaired bone homeostasis and could contribute to varying response to treatment. We present the case of two brothers with early osteoporosis who were found to have a heterozygous variant of unknown significance in the *WNT1* gene, c.1060_1061delCAinsG (p.H354Afs*39). This finding demonstrates that frameshift variants in *WNT1* may also act in a dominant fashion leading to decreased bone mass.

## 1 Introduction

Osteoporosis is characterized by reduced bone mineral density (BMD), impaired bone quality with high predisposition to fractures. It is a multifactorial disease that results from complex interactions between metabolic, genetic, and environmental factors, with several underlying mechanisms that have not been completely elucidated (Al Anouti et al., 2019).

The WNT signaling pathway is recognized as an essential regulator of the bone remodeling process (Baron and Kneissel, 2013). It is subdivided into three signaling processes: the canonical WNT pathway, the noncanonical WNT-planar cell polarity pathway and the WNT-calcium pathway. Through multiple complex interactions and regulations, activation of the WNT signaling pathway leads to increased bone formation and reduced bone resorption. Genetic alterations of this pathway resulting in loss-of-function in WNT signaling lead to reduced bone mass and high risk of fracture. On the contrary, alterations resulting in gain-of-function are associated with increased bone mass (Baron and Kneissel, 2013).

Loss-of-function variants in *WNT1* have been described in cases of an autosomal recessive form of osteogenesis imperfecta (Laine et al., 2013), which is caused by impaired bone formation. A heterozygous *WNT1* pathogenic variant has been also seen in patients with early-onset osteoporosis (Luther et al., 2018).

We present the case of two brothers with early-onset osteoporosis who were found to have a heterozygous variant in *WNT1.* Although reported as variant of unknown significance (VUS) by clinical genetic testing laboratory based on ACMG (American College of Medical Genetics) criteria, given the clinical presentation, location of the variant and known importance of WNT pathway in bone formation, we suggest that early bone loss could be related to this variant. To our knowledge, the variant in the *WNT1* gene that we found in these brothers has not been reported previously.

## 2 Case description

### 2.1 Brother 1 (II.3)

A 72-year-old man with history of obstructive sleep apnea who presented for management of osteoporosis. He suffered from multiple spontaneous rib fractures at age 35 while skiing. When he turned 40 years old, he experienced back pain while working as a carpenter lifting heavy weighs, found to have T7 compression fracture. He was treated with oral alendronate for 10 years until age 50.

**Fig. 1:**
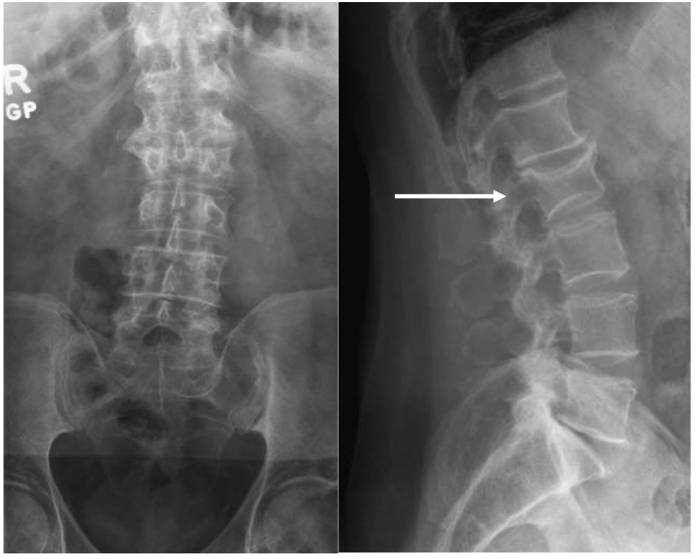
Radiography of the lumbar spine A) antero-posterior view B) lateral view. White arrow denotes compression fracture of L2.

At age 66, he developed new non-radiating lower back pain while running on a track. He was diagnosed with an L2 compression fracture (Fig. 1) and was referred to Endocrinology at the Philadelphia Veterans Affairs Medical Center for further management. A Lunar Prodigy dual-energy X-ray absorptiometry (DXA) showed a BMD of 0.815 g/cm^2^ with T-score of −3.3 in L3-L4 spine (L2 excluded due to compression fracture). Workup of secondary causes of osteoporosis (which included measurement of phosphorus, intact parathyroid hormone, 25-hydroxyvitamin D, serum protein electrophoresis, testosterone, and urinary calcium) was negative except for genetic testing (*WNT1* variant) described below. He received teriparatide 20 μg daily for 2 years with significant improvement in BMD at L3-L4 to 1.010 (+20%) with T-score -2.0.

**Fig. 2:**
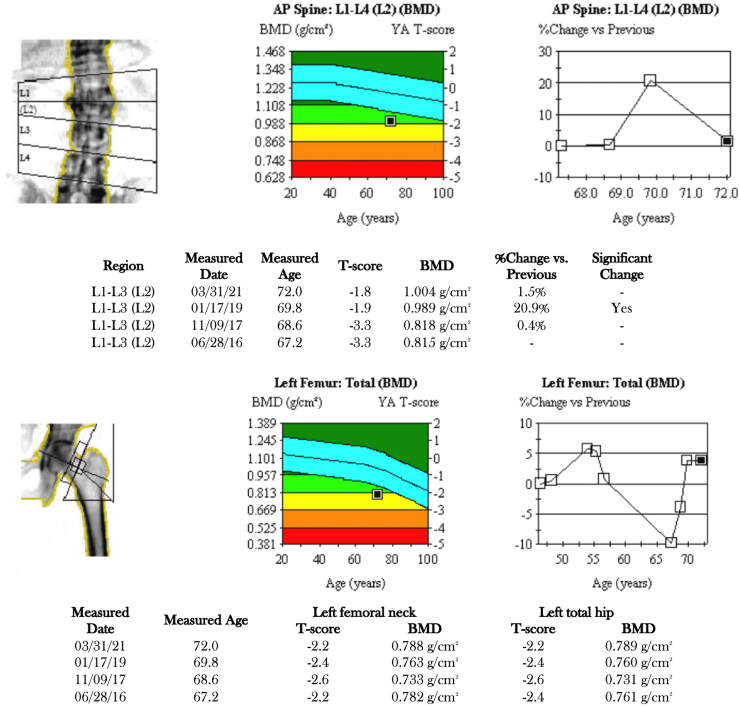
Results of dual-energy X-ray absorptiometry (DXA) from 2016 to 2021 of Brother 1. Bone density measurements of lumbar spine (except for L2), left femoral neck and total hip were obtained using the Lunar Prodigy Advance DXA system. He received teriparatide from 2016 to 2018, followed by denosumab since 2019.

After completing treatment with teriparatide, he began treatment with denosumab 60 mg every 6 months, which is ongoing. At age 71, the patient reported right foot pain while walking which improved over time. X-ray and computed tomography confirmed fracture of second metatarsal base. Repeat DXA scan showed sustained improvement in BMD at L3-L4 to 1.032 (+ 1.5%) with T-score -1.8 (Fig. 2).

### 2.2 Brother 2 (II.5)

A 64-year-old man with history of melanoma and hypercalciuria was referred to Penn Bone Center for management of osteoporosis at age 58. The patient had experienced multiple fractures since age 10 including femur, wrist, ribs, skull, and toe fractures, which resulted from trauma such as playing soccer or falling from a bike. He was diagnosed with osteoporosis when he was 48 years old. Prior to establishing care at our center, he had received treatment with oral alendronate for 6 years until age 54, transitioned to yearly intravenous zoledronic acid due to decline in BMD at the hip. Received four doses of zoledronic acid, last dose was given when he was 58 years old.

**Fig. 3:**
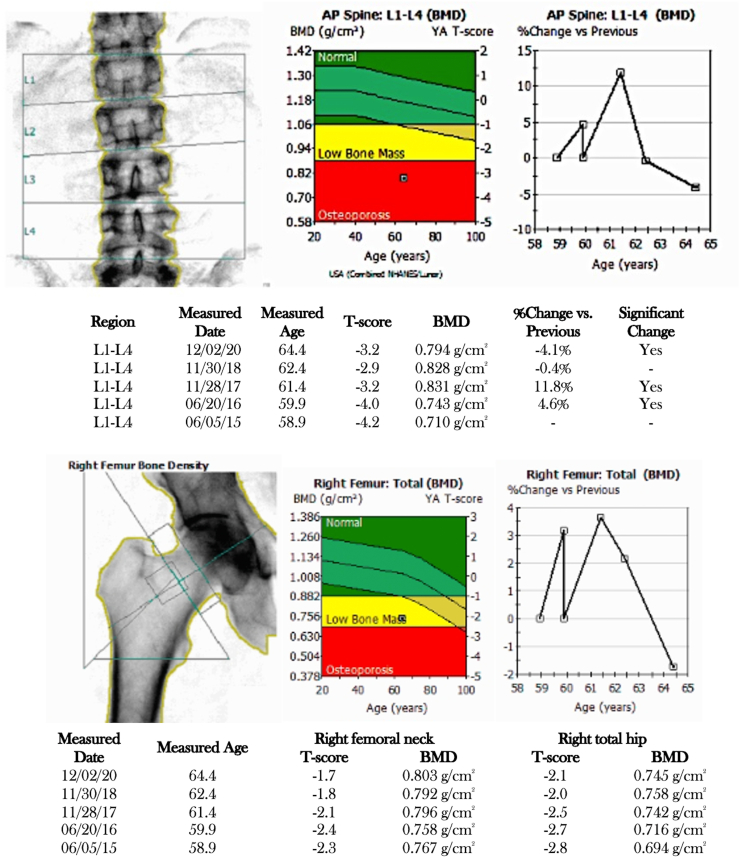
Results of dual-energy X-ray absorptiometry (DXA) from 2015 to 2020 of Brother 2. Bone density measurements of lumbar spine, femoral neck and total hip were obtained using the Lunar DXA system. He received teriparatide from 2016 to 2018, followed by one dose of zoledronic acid in May 2019.

The patient showed minimal improvement in BMD after 6 years of oral alendronate and 4 years of intravenous zoledronic acid (Fig. 3). Prior secondary evaluation for osteoporosis was unremarkable except for hypercalciuria (urinary calcium 340 mg/24 h), for which he was treated with thiazide diuretics. He also had a skin biopsy for procollagen I and III which was negative.

At age 60, the patient tripped at home and fractured his left wrist requiring surgical repair. Due to ongoing fractures, no improvement in BMD with bisphosphonates, and concern for long term side effects associated with bisphosphonates, osteoanabolic therapy with teriparatide was initiated. His urinary calcium was monitored over time while on teriparatide, which remained stable on thiazides and cross-sectional renal imaging was negative for nephrolithiasis. He had a significant improvement of 12% in BMD as detailed in Fig. 3.

The patient completed a 2-year course with teriparatide, followed by one intravenous zoledronic acid. Did not experience any further fractures and BMD remained overall stable with slight decline in spine. Plan is to repeat DXA in 1 year and consider romosozumab if there is further decline in BMD or if he sustains additional fractures. In terms of secondary causes of osteoporosis, given strong family history of osteoporosis, patient was referred to see Genetics for second opinion, found to have variant in the *WNT1* gene (described below).

### 2.3 Family history

**Fig. 4:**
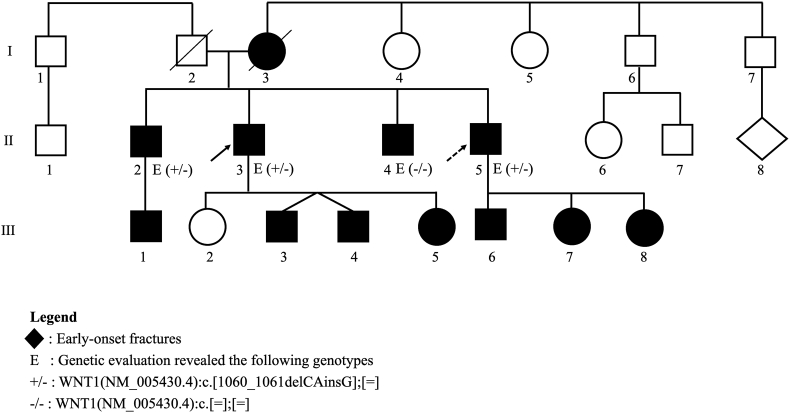
Pedigree diagram**.** Brother 1 (II.3) is pointed to by a solid arrow and brother 2 (II.3) is pointed to by a dashed arrow. Members with fractures at a young age are colored in black. Members who had genetic evaluations are marked with E (+/−) if the variant was detected or E (−/−) if it was absent.

A summary of the family history has been represented in a genetic pedigree in Fig. 4. Our patients are denoted by arrows. Their mother (I.3) was of Hungarian descent. She suffered from a vertebral fracture and multiple rib fractures at age of 52. She died at age 94. Their father (I.2) was of Irish descent. He did not have history of fractures, osteoporosis, parathyroid problems, or kidney stones. He died at age 89.

Our patients have two brothers. Oldest brother (II.2) (also found to have *WNT1* variant) is 74 years old. He was diagnosed with osteoporosis at age 55; he suffered an ankle fracture while playing soccer at age 61 and has been treated with oral alendronate. He has one son (III.1) who is 25 years old. He suffered an arm fracture during car accident.

Another brother (II.4) is 69 years old. He tested negative for *WNT1* variant. He had history of osteopenia, suffered an ankle fracture at age 51. His DXA scan showed a BMD (T-score) of 1.001 g/cm^2^ (−1.5), 0.929 g/cm^2^ (−0.8), and 0.933 g/cm^2^ (−0.6) at the lumbar spine, right femoral neck and right total hip, respectively. He has been treated with bisphosphonates.

Brother 1 (II.3) has four descendants, three of them (III.3, III.4, III.5) have experienced fractures from level-level trauma. Brother 2 (II.5) has 3 descendants (III.6, III.7, III.8), all of them have experienced fractures from low-level trauma as well. None of their children have undergone genetic testing.

### 2.4 Genetic testing

Gene panel testing was completed for each patient (brother 1 [II.3] and brother 2 [II.5]). Genomic DNA from saliva sample was used for analysis of a selected panel of genes associated with low BMD via exome slice. The exonic regions and flanking splice junctions of the genome were captured using a proprietary system developed by GeneDx and sequenced by massively parallel (NextGen) sequencing on an Illumina sequencing system with 100 bp or greater paired-end reads. Reads were aligned to human genome build GRCh37/UCSC hg19 and analyzed for sequence variants in the selected genes or regions of interest using a custom-developed analysis tool (Xome Analyzer).

Other genes that were evaluated with no identification of other variants were: *ALPL, ANKH, AP2S1, BMP1, CASR, CLCN5, COL1A1, COL1A2, CREB3L1, CRTAP, CYP27B1, CYP2R1, DMP1, ENPP1, FAH, FAM20C, FGF23, FKBP10, GNA11, IFITM5, OCRL, P3H1, PHEX, PLOD2, PLS3, PPIB, SERPINF1, SERPINH1, SLC34A1, SLC34A3, SLC9A3R1, SP7, SPARC, TMEM38B, VDR*.

**Fig. 5:**
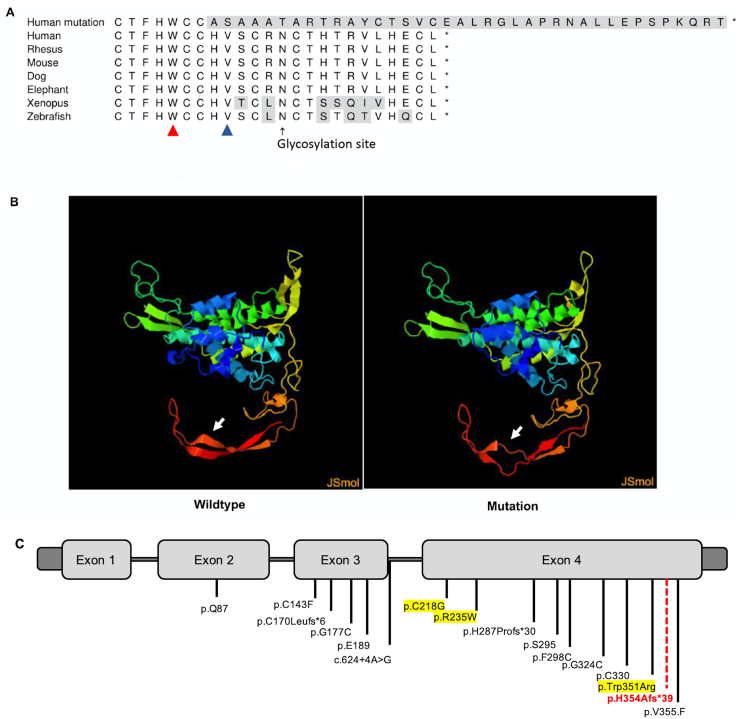
*WNT1* variants. A) Alignments based on the UCSC genome browser and Homologene. Glycosylation site from Uniprot. Arrow heads indicate previously reported pathogenic variant causing dominantly inherited early onset osteoporosis (Red arrow head, p.Trp351Arg) and recessively inherited osteogenesis imperfecta (Blue arrow head, p.Val355Phe) B) 3D modeling using Phyre2 based on PDB structure entry 7DRT for Wnt3a**.** C) Representation of the gene structure of *WNT1* indicating previously published pathogenic variants and their positions (not drawn to scale). Variants highlighted in yellow are transmitted in an autosomal dominant pattern. Dashed red line denotes position of novel variant (p.H354Afs*39). (For interpretation of the references to colour in this figure legend, the reader is referred to the web version of this article.)

Each brother (II.3 and II.5) was found to have a heterozygous variant in the *WNT1* gene, c.1060_1061delCAinsG (p.H354Afs*39) (Fig. 5) Following this, their two additional brothers (II.2 and II.4) underwent single-site sequencing to evaluate for this variant. II.2 was found to have the variant while II.4 was not. The analysis of the variant by clinical genetic testing laboratory based on ACMG criteria was classified as VUS: PM2 (not observed at significant frequency in large population cohorts), PM4 (frameshift variant predicted to result in protein truncation as the last 17 amino acids are replaced with 38 different amino acids, although loss-of-function variants have not been reported downstream of this position in the protein), PP4 (identified in a patient with personal and family history consistent with childhood onset osteoporosis, referred for genetic testing at GeneDx).

## 3 Discussion

We report the case of two brothers with early onset osteoporosis, who were found to have a heterozygous variant in the *WNT1* gene, c.1060_1061delCAinsG (p.H354Afs*39). The c.1060_1061delCAinsG variant has not been reported previously as a pathogenic variant nor as a benign one. It causes a frameshift starting with codon Histidine 354 which changes this amino acid to an Alanine residue, and creates a premature Stop codon at position 39 of the new reading frame (p.His354Alafs*39). This variant is predicted to replace the last 17 amino acids of the protein with 38 incorrect amino acids (Fig. 5A). The analysis of human *WNT1* compared to orthologous proteins in vertebrates shows good conservation of the C-terminal amino acids (Fig. 5A). Our analysis using Phyre2 predict that this conserved protein's secondary and tertiary structure would be changed by the mutation (Fig. 5B). Moreover, it would lead to the loss of a glycosylation site, and the loss of a Valine affected by a mutation in a recessive OI family previously reported. This variant is in exon 4 of the *WNT1* gene, which contains most of the previously reported pathogenic variants (Fig. 5C). Two pathogenic variants causing osteogenesis imperfecta and early onset osteoporosis are located near this variant (Fig. 5B, (Fahiminiya et al., 2013, Alhamdi et al., 2018). Although this variant is classified as VUS by the clinical genetic testing laboratory based upon ACMG criteria, these findings are suggestive of pathogenicity. These results suggest that the variant within the *WNT1* gene is likely damaging and contributing to early-onset osteoporosis in our patients.

Interestingly, one of the four brothers with osteopenia tested negative for this variant, which could explain his mild degree of bone loss. This could suggest additional genetic modifiers impacting bone density, including possible variants in genes that interact with *WNT1*, within this family. Although their descendants have not been tested for this genetic variant yet, a number have had low-impact fractures as well. Genetic testing for these individuals along with bone density evaluations could help understand the impact of this variant better.

Osteoporosis is a silent disorder characterized by decreased bone strength leading to increased risk of fractures. Although it is more prevalent in women, men with hip and vertebral fractures have a higher mortality rate (Haentjens et al., 2010). Secondary causes can be identified in the majority of cases and previous studies have shown that genetic defects play an important role in the pathogenesis of men with idiopathic osteoporosis (Van Pottelbergh et al., 2003). In the case of our two patients, an underlying genetic cause was suspected given early-onset osteoporosis and fragility fractures seen across generations.

Several genes have been recognized as being involved in the pathogenesis of osteoporosis (Ralston and Uitterlinden, 2010; Estrada et al., 2012; Morris et al., 2019; Trajanoska and Rivadeneira, 2019); however, only few cases have been reported of *WNT1* pathogenic variants (Laine et al., 2013; Kausar et al., 2018). WNT1 serves as a ligand in the canonical WNT signaling pathway, which is essential for normal bone metabolism, required for osteoblast differentiation and bone formation. WNT1 promotes bone formation by binding to the LRP5-Frizzled receptor complex and activating the canonical WNT signaling pathway. *WNT1* pathogenic variants lead to decreased WNT1 signaling which results in bone fragility.

In this family, osteoporosis seems to be inherited in an autosomal dominant manner. Pathogenic variants in *WNT1* have been implicated in both autosomal recessive osteogenesis imperfecta type XV and in autosomal dominant early-onset osteoporosis. The variants previously reported in autosomal dominant osteoporosis were in exon 4 as well (highlighted in yellow in Fig. 5) (Keupp et al., 2013; Laine et al., 2013; Alhamdi et al., 2018).

Patients with *WNT1* variants and bone loss have shown no substantial response to treatment with bisphosphonates (Keupp et al., 2013), similar to what we observed in our patients. The lack of response to this class of medications is in line with the dysfunction of osteoblasts which results from reduced WNT1 signaling. Understanding the pathogenesis and role in bone metabolism of the *WNT1* variant could facilitate the use of more effective medical therapies for patients with osteoporosis.

Our study had several limitations. Additional family members declined both clinical evaluation and variant testing. Therefore, we were unable to obtain results of DXA scan or to perform genetic testing on any other relatives. Additionally, we were unable to use functional assays to test for pathogenicity of this variant.

## 4 Conclusions

A previously unreported variant in the *WNT1* gene was identified in this family with early onset osteoporosis. Although reported as VUS, the clinical presentation, location of the variant, and known importance of WNT pathway in bone formation support that early bone loss could be related to this variant.

## Funding source

This research did not receive any specific grant from funding agencies in the public, commercial, or not-for-profit sectors.

## CRediT authorship contribution statement

Authors have contributed to the conceptualization and design, writing of original draft, review, editing, and approval of final version.

## Declaration of competing interest

None
